# Food’s future: sustainability and agricultural robotics

**DOI:** 10.3389/frobt.2025.1696483

**Published:** 2026-04-07

**Authors:** Sindiso M. Nleya, Siqabukile Ndlovu, Mthulisi Velempini

**Affiliations:** 1 Computer Science Department, University of Limpopo, Polokwane, South Africa; 2 Computer Science Department, National University of Science and Technology, Bulawayo, Zimbabwe

**Keywords:** agricultural robotics, artificial intelligence, automation, climate resilience, food security, precision agriculture, resource optimization, sustainability

## Abstract

Our global food system faces growing challenges such as population growth, climate change, resource constraints, and food loss. This set of threats has begun to erode the stability of food security efforts and challenge the long-term sustainability goals outlined by global organizations. To respond effectively, the sector needs concrete and forward-looking innovations that reflect the objectives of the Sustainable Development Goals (SDGs) of the United Nations (UN), especially the commitment in Goal 2 to eliminate hunger. In this study, we examine how agricultural robotics can support the shift toward more resilient and sustainable food systems, particularly in areas where classical methods are under strain. It brings together perspectives from technology, sustainability, and policy, aiming to bridge broad global priorities with everyday realities faced in local contexts. To structure the discussion in a concise way, our analysis is framed around five different, yet interrelated, dimensions. First, we use a crisis-framing perspective to explain why food system reform has become urgent and to show how these pressures align with key SDG priorities. The second dimension outlines a simple taxonomy that groups agricultural robots according to their domain and intended function while also highlighting ongoing technical issues such as interoperability. The next dimension examines how robotics is being amalgamated with precision farming tools, Internet of Things (IoT) platforms, artificial intelligence (AI), and big data systems. Collectively, these technologies facilitate more autonomous field operations and support faster, data-driven decision making. The sustainability dimension evaluates how these technologies affect environmental, economic, and social outcomes in the agricultural sector. This comprehensive review highlights several potential advantages, such as reduced chemical inputs, improved water efficiency, improvements in soil quality, more efficient use of labor, and new employment opportunities in rural and remote areas. In the final dimension, this study turns to global case studies, drawing comparative insights between developed nations such as Australia and the United States, and emerging economies including Brazil, India, and China. Across these diverse contexts, agricultural robotics consistently demonstrate the capacity to boost productivity, reduce waste, and make more efficient use of resources. It is apparent that these gains extend beyond the farm, contributing to environmental stewardship and broader socio-economic development. Yet, the path to widespread adoption is far from straightforward. Farmers and policymakers alike confront persistent barriers: the high upfront costs of robotic systems, gaps in technical expertise, difficulties in ensuring interoperability across platforms, and pressing ethical questions around data governance and automation. Overcoming these challenges is not simply a technical exercise; it is a prerequisite for realizing the full promise of robotics in reshaping global food systems for a more sustainable future.

## 1 Introduction

Population growth, climate change, food waste, food shortages, and resource limitations represent interconnected challenges to the international food system that threaten food security and sustainable progress (World Bank, 2022; World Bank, 2025; Fu et al., 2025). The global food and nutrition environment is deteriorating year by year, with increasing levels of malnutrition. In 2023, approximately 733 million people suffered from malnutrition—152 million more than in 2019. To make matters worse, a secondary but critical issue is at play as the “hidden hunger” phenomenon indicates that in 2022, 2.8 billion people globally lacked access to a healthy diet due to increasing food prices and more pronounced income gaps. The vulnerability of populations to food price shocks was starkly illustrated that same year, when World Bank data revealed that a 1% increase in international food prices pushed an extra 10 million people into abject poverty. From an economic standpoint, researchers at the University of Oxford and the London School of Economics estimate that market failures and systemic inefficiencies generate 10 trillion in hidden costs annually within the global food system. Meanwhile, the Office for the Coordination of Humanitarian Affairs (OCHA) (Bank, 2025) underscores the scale of acute food insecurity, reporting that over 280 million people face severe hunger daily.

Their examination underscores the profound interconnectedness between food insecurity and wider humanitarian emergencies and highlights the acute need for an integrated international response. To sustainably nourish populations on a livable planet, the inefficiencies and losses present within the global food system reflect the urgent need for systemic change. Increasing efficiency, equity, and minimizing waste are central to building resilient and inclusive food systems. Without these bold and coordinated investments, approximately 950 million people could be at risk of extreme food insecurity by 2030, placing us further from the universal goal of Zero Hunger (Kannan, 2025; Wah, 2025; Ray et al., 2024). Manifestly, harnessing technological innovation to address global hunger is pivotal to advancing sustainable agriculture, an essential pillar for achieving Goal 2: Zero Hunger. Technology transforms food systems by supporting efficient, sustainable, and economically resilient food production (Hiywotu, 2025). Sustainable agriculture is defined as “an integrated system of animal and plant production practices with site-specific applications that will (over the long term): (a) meet human food and fiber requirements; (b) improve environmental quality; (c) make effective use of non-renewable and on-farm resources while integrating natural biological cycles and controls; (d) sustain the economic viability of farm operations; and (e) enhance the quality of life for farmers and society as a whole” (Velten et al., 2015). Achieving sustainable agriculture on a global scale requires the adoption of advanced technologies that enhance efficiency, minimize waste, and optimize resource use. Among these innovations, robotic technology has emerged as a transformative solution. By automating labor-intensive tasks, improving precision in resource application, and enabling real-time crop monitoring and management, robotics contributes significantly to agricultural productivity and long-term sustainability (Anya et al., 2024). While numerous studies in agricultural robotics have focused on automation for yield optimization (Barrile et al., 2025; Hamrani et al., 2025), labor reduction (Marinoudi et al., 2019; Bogue, 2024), and precision farming (Gangwani, 2024), fewer have examined the synergistic integration of robotic systems with sustainability principles, particularly in relation to energy efficiency, resource conservation, and ecosystem resilience. The unique contribution of this work lies in its multidimensional and regionally contextualized approach to agricultural robotics as a strategic catalyst for sustainable food systems. Subsequently, this study offers a holistic synthesis of technology, sustainability, and policy, examined through both global and local perspectives. While classical agricultural practices hold cultural significance and may be well suited to specific local contexts, they often face limitations in meeting broad sustainability objectives. These constraints span environmental, social, and economic dimensions, making it imperative to complement traditional methods with modern, scalable solutions (Boros et al., 2024).

### 1.1 Key contributions

The main contribution of this study is its regionally grounded, multidimensional examination of how agricultural robotics can support more sustainable food systems. Rather than treating the technology in isolation, the study connects technological advances with sustainability goals and policy considerations, showing how global priorities interact with the practical realities faced in local farming contexts. First, this study frames the global food crisis by quantifying systemic challenges such as increasing population, changing climate, malnutrition, and resource scarcity, linking these directly to the UN Sustainable Development Goals, particularly Goal 2: Zero Hunger. Second, it presents a comprehensive taxonomy of agricultural robotics, classifying robotic platforms by domain, task, mobility, control mechanisms, and specialization while addressing technical challenges such as interoperability and robustness under real-world farm conditions. Third, the study examines the integration of emerging technologies, including precision agriculture, IoT, sensor networks, AI, machine learning, big data analytics, and GPS, highlighting how their convergence enables predictive analytics, autonomous operations, and real-time decision-making. Fourth, it evaluates the contribution of agricultural robotics to environmental, economic, and social sustainability, demonstrating impacts such as reduced chemical use, improved water efficiency, minimized soil compaction, improved yield and quality, reduced labor dependency, and rural job creation. Fifth, this study presents global case studies from China, Brazil, Germany, Canada, India, and Australia, showcasing diverse applications and measurable outcomes. For instance, Brazil’s Solix robot achieved a 95% reduction in herbicide use and scalable deployment. Finally, the study identifies persistent barriers to adoption, including huge startup expenses, complex technical challenges, lack of interoperability, and ethical concerns, and offers policy recommendations such as open-source platforms, regulatory harmonization, farmer training, and public–private partnerships to support inclusive and scalable innovation.

### 1.2 Study organization

Beyond the introduction (Section 1), this study comprises eight other distinct sections, each contributing to a coherent exploration of agricultural robotics within the broader context of sustainable development. Section 2 presents the methodological approach to the study detailing the research design, along with the baseline and effect-size analysis. This is followed by Section 3, which focus on the imperative of sustainable agriculture, wherein an exposition of the sustainability dimensions is introduced. Section 4 is a comprehensive taxonomy of agricultural robots, classifying them by function, mobility, and control architecture. Section 4 delves into the technological foundations, including sensor integration, artificial intelligence (AI) algorithms, and energy systems. Section 5 explains the role of robotics in sustainability. Section 6 focuses on case studies from both developed and emerging economies, along with associated insights. Section 7 identifies key barriers to adoption, including technical, financial, and regulatory, and analyzes their implications. Section 8 proposes strategic pathways for scaling robotics, focusing on open-source innovation, stakeholder collaboration, and policy reform. Finally, Section 9 synthesizes the findings and aligns them with the UN Sustainable Development Goals, offering a visionary outlook on the future of agricultural automation.

## 2 Methodology

The study leverages the enhanced methodology (Zhang et al., 2024). The enhanced methodology entails a combination of published studies on a particular subject matter with the intention of comprehensively understanding the topic. Specifically, as described by Nleya and Velempini (2024), we strive to determine the consequences of agricultural robotics for food production and sustainability.

### 2.1 Research design

**FIGURE 1:**
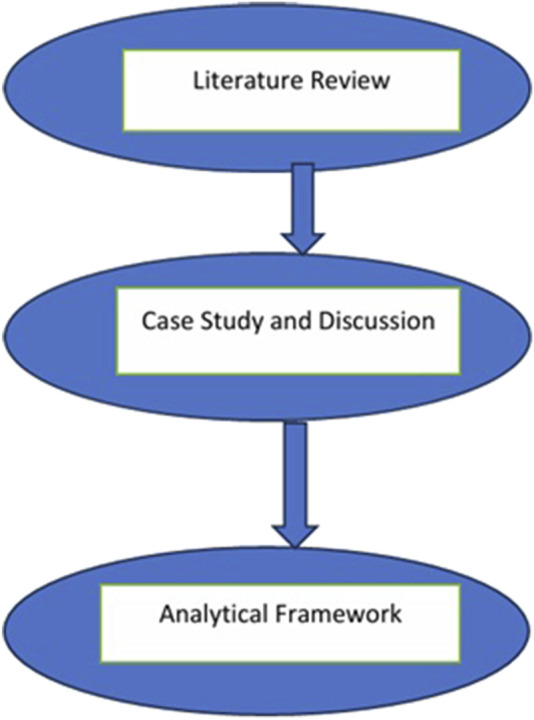
Research design.

Practically, this study adopts a three-phased approach, as illustrated in Figure 1. It elucidates how articles in our analysis of agricultural robotics and its influence on food production systems and sustainability were chosen. Initially, our literature search strategy involved a systematic search across databases such as Web of Science, Scopus, and AGRICOLA. In the process, we used keywords such as “agricultural robotics,” “sustainability,” “precision agriculture,” “food systems,” “automation,” and “climate resilience.” Several sources of articles and literature were encountered; thus, a search inclusion criterion was applied. The inclusion criteria considered peer-reviewed articles published in the last 10–15 years, studies linking robotics to at least one sustainability pillar, and those containing empirical data or robust modeling. Certainly, our exclusion criteria encompassed non-agricultural robotics, opinion pieces lacking technical depth, and studies without geographic or contextual relevance. In the second stage, we considered case studies wherein our selection strategy involved applying a purposeful sampling approach (Rübcke von Veltheim and Heise, 2021). The typical cases include countries in two categories.

- Developed economies, defined as countries with mainstream adoption (e.g., United States and Australia).
- Emerging economies (e.g., India and Brazil).

To this end, our criterion first included the availability of data across the three sustainability pillars (environmental, economic, and social). Second, a fair representation of different agroecological zones was considered, and finally, the policy relevance and scalability of robotic interventions were also considered. In the third stage of the analytical framework, the coding scheme leveraged the pillar-based matrix. Subsequently, a comparative analysis is performed in which the findings are cross-tabulated by country and technology type. To do this, we identify patterns, gaps, and outliers. In the validation aspect, this stage triangulates findings through academic sources, policy reports, and industry data. As a result, reflexive analysis is applied to account for bias and limitations in the data.

#### 2.1.1 Comparative baseline and effect-size analysis

Percent change results were referenced to a universal baseline for inter-study comparability. For mean values with a standard deviation or n, either n or standard deviation was chosen to calculate effect size and a 95% confidence interval. Therefore, a meta-analysis relative to the impact of robotics on sustainability is valid, albeit with greater heterogeneity due to crop type, farm size, and agro-climatic region.

This approach establishes baseline studies that allow for counterfactual comparisons in agricultural robotics—for example, evaluating yield, water use, emissions, or soil health against traditional farming methods. The broader idea is that robotics can be assessed in measurable, real-world terms, making it possible to develop policies and investment strategies that are grounded in clear environmental and economic evidence.

## 3 Imperative for sustainable agriculture

**FIGURE 2:**
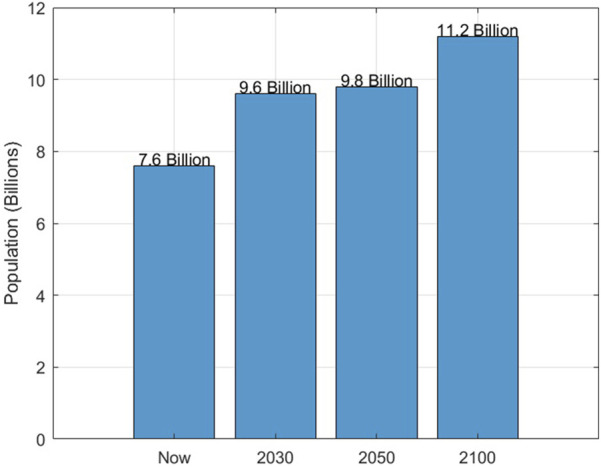
Population projection.

The world population, estimated at approximately 7.6 billion, is anticipated to increase, reaching 8.5 billion by 2030 , climbing to 9.8 billion in 2050 , and finally peaking at 11.2 billion in 2100 , as depicted in Figure 2. Significantly, developed and emerging economies are projected to experience a disproportionately high population increase.

**FIGURE 3:**
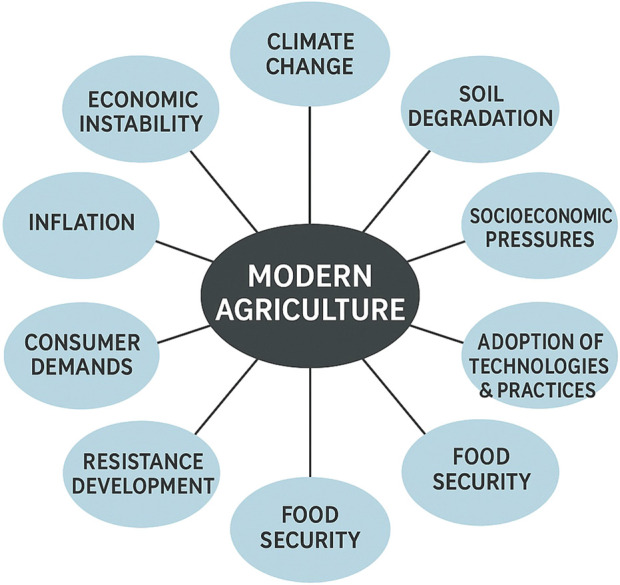
Modern agriculture.

Furthermore, according to the push–pull theory of migration (Ghodsi et al., 2024), populations tend to move from rural to urban areas (Mthiyane et al., 2022) for better living standards. Finite resources will be subjected to extreme pressure in relatively smaller areas, further putting the economy at risk. Subsequently, this worsens the challenges related to sustainable agricultural production and land management. According to Suchithkumar et al. (2024), modern agriculture faces numerous challenges, as shown in Figure 3, such as climate change, soil management, and the critical need to maintain and enhance global soil biodiversity. In addition, the population surge and urbanization significantly reduce the available land for cultivation. This raises the crucial question of dietary health and food security for the expanding global population. Furthermore, changing lifestyles contribute to the growing issue of malnutrition. In summary, modern agriculture faces a multitude of interconnected challenges, including the results of climate shift; soil degradation (erosion, declining water levels, and biodiversity loss); socioeconomic pressures (increasing population, urbanization, migration, labor availability, land shrinkage, and investment); productivity concerns; difficulties in adopting new technologies and management practices; and critical issues related to food security, malnutrition, evolving consumer demands, resistance development, inflation, and global economic instability.

Sustainable agriculture management in European Union countries (DeLonge et al., 2016) defines sustainable agriculture through a spectrum of practices that support socio-ecological food systems. The categories relevant to this analysis are as follows:

1. Boosting efficiency to minimize input usage (L1).
2. Integrating sustainable alternatives into agricultural practices (L2).
3. Reconfiguring systems using ecological principles (L3: agroecology).
4. Fostering direct ties between producers and consumers as a foundation for socio-ecologically redesigning the food system (L4: social dimensions of agroecology).
5. A fifth dimension of agricultural sustainability, as presented by Gliessman (2021), emphasizes a participatory, equitable, and just food system based on L3 and food links, anchored by L4 (Pennsylvania Envirothon, 2019).

**FIGURE 4:**
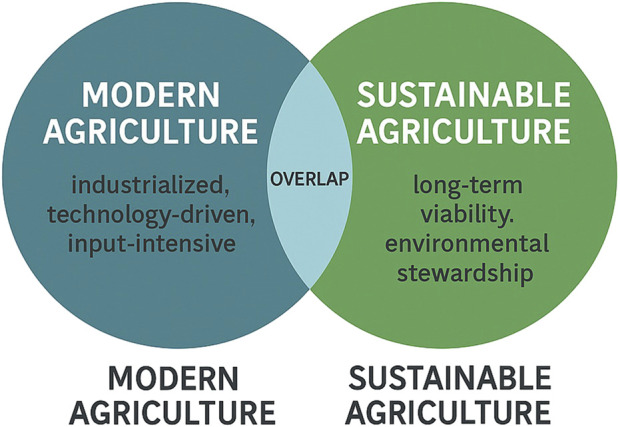
Modern agriculture and sustainable agriculture overlap.

As a result, there is general overlap between modern agriculture and sustainable agriculture, as depicted in Figure 4.

## 4 Agricultural robotics: an overview

The multifaceted agricultural landscape is rapidly evolving, driven by the convergence of robotics, AI, machine learning (ML), and data-centric technologies. The agricultural robotics paradigm represents a transformative shift from manual labor to intelligent automation, enabling farmers to meet growing global food demands with greater precision, efficiency, and sustainability. Robotic platforms—ranging from autonomous tractors and harvesters to aerial drones—are now integral to precision agriculture, where targeted interventions optimize crop yield and resource use. At the core of this revolution lies data. Sensors embedded in fields and machinery continuously collect real-time data on soil health, moisture levels, crop growth, and pest activity. These data, when processed through AI and ML algorithms, empower predictive analytics and informed decision-making. Big data platforms aggregate and analyze vast datasets, revealing patterns that were previously invisible to the human eye. Coupled with Global Positioning Systems (GPS), these technologies enable hyper-localized actions, from variable rate irrigation to automated weeding—tailored to the specific needs of individual field plots. This section explores the foundational components of agricultural robotics, highlighting how integrated systems are reshaping farming into a high-tech, data-driven enterprise. From field to cloud, the fusion of robotics and intelligent technologies is redefining what it means to cultivate the land.

- Robotic platforms: An agricultural robotic platform is a portable system, typically with mechanical structures and electronic components, designed to perform multiple farming operations autonomously or with little human interaction. These platforms are used in different agricultural settings and range from small, single-operation robots, designed for tasks such as weeding or harvesting, to larger, multipurpose robots capable of performing operations such as planting, spraying, and field preparation (Garcia et al., 2019). The main features of robotic platforms are described in the robotic platform use (Sotnik and Lyashenko, 2022), such as soil preparation, planting, watering, field monitoring, harvesting, spraying, pest control (sprayers), and automated multifunctional platforms. These are different approaches to the classification of robotic platforms; one approach, presented by Duckett et al. (2018), classifies robots by task and domain. Robots are engineered to carry out a single, defined task on a particular crop within an agricultural environment. An alternative approach, proposed by Sotnik and Lyashenko, (2022), classifies robots based on their functions and applications in farming. These encompass the type of work, the nature of movement, the type of control, the level of specialization, the agricultural environment in which they are used, and the operating conditions. Generally, field farms have variable infrastructure, implying that early robot machines could only perform tasks in specific field farms and not in all fields. Thus, most existing agricultural robots struggle in real-world environments as they are unable to reliably operate in conditions such as mud, fog, rain, or extreme temperatures. For instance, many robotic manipulators cannot withstand the high humidity in greenhouse environments.
- Precision agriculture (PA): a technology-enabled, data-inspired approach to farming management that involves observing, measuring, and analyzing the requirements of individual crops and fields. It can support the broader aim of meeting increasing food demand while maintaining sustainable primary production through a more complex, precise, and resource-efficient approach to managing agricultural processes. PA is categorized into two types: first, precision-based crop farming, comprising the utilization of precision agricultural technologies for the management of changes across location and over time for improvement of crop production and environmental context; and second, precision livestock farming (PLF), leveraging advanced technologies to optimize the performance of individual animals.

PA is intertwined with robotics, with PA serving as the strategic brain and agriculture robots as the operational muscle. According to Agricial (2023), PA has a strategic impact as it promotes efficiency, where robots perform tasks faster and more accurately when guided by precision data. Additionally, PA promotes sustainability, where targeted interventions reduce chemical use, fuel consumption, and soil degradation. Finally, it facilitates scalability when PA systems allow robotics to adapt across diverse terrains and crop types.

- Internet of Things (IoT): IoT basically refers to connecting any device or object to the Internet and enabling them to send or receive data (Dhanwe et al., 2024). Applied to robotics, it enables smart, autonomous machines that can communicate, collect, and analyze data and make decisions independently. The synergy between IoT and robotics has subsequently opened up possibilities and opportunities in different domains. In the agricultural domain, IoT has reorganized the approach to farming by transforming classical farming into a dynamic, data-driven, and sustainable system tailored to meet the growing global food demand (Duguma and Bai, 2024; Padhiary et al., 2025). IoT integrates sensor technologies, devices, and analytics to unlock opportunities for PA, optimization of resources, and predictive decision-making. IoT is the nervous system of agricultural robotics—it connects, informs, and empowers robotic systems to operate intelligently and sustainably.
- Data-driven: Revenue, efficiency, productivity, and profitability can all be considerably raised in the agricultural value chain by implementing data-driven robotics systems. Data collection in soil, weather, crops, animals, machinery, and equipment is made possible by robotic systems that are equipped with numerous sensors, sample tools, and GPS autopilots. AI/ML can further process and analyze these raw data to provide insightful information that helps farmers make better decisions and provide higher-quality goods and services.
- Artificial intelligence: Artificial intelligence has revolutionized. The use of AI in agricultural robotics is transforming contemporary farming methods, resulting in greater production, sustainability, and efficiency. AI has numerous applications in agriculture, such as irrigation, weeding, and spraying, facilitated by sensors and other technologies embedded in robots and drones. These technologies reduce the overuse of pesticides, water, and herbicides; maintain soil fertility; and help efficiently use manpower while increasing productivity and quality (Talaviya et al., 2020). By leveraging AI-based technologies, farmers can improve productivity, reduce inputs, improve quality, and access markets more quickly. Today, the world’s population, the reduction of arable land, and the shortage of workers in the agricultural sector require robotic agriculture. The system can undergo a long lasting transformation due to robotic agriculture.

With the correct sensors, agricultural robots can save money, promote sustainability, and reduce their carbon footprint (Hokuyo USA, 2025a). AI technologies, especially in ML, play an important role in most of the above technology areas and will be vital for agricultural robots (Duckett et al., 2018).

- Machine learning: ML is a branch of AI that enables computers and machines to mimic the human ways of learning, autonomous task performance, and improvement of performance and accuracy by way of experience and exposure to more data (IBM, 2021). ML is transforming agricultural robotics by enabling robots to perform tasks such as crop monitoring, weed detection, and harvesting with greater precision and efficiency, eventually leading to improved yields and optimized resource use. Robots can identify patterns in vast volumes of data using machine learning techniques. Robots can improve their performance on tasks through machine learning. In essence, it teaches students to improve over time rather than only adhering to preset instructions. Robots obtain data from their environment using sensors such as cameras and microphones. They then train machine learning algorithms using these data. These models assist robots in identifying patterns, drawing conclusions, and acting on their acquired knowledge (Standard, Bots, 2025). There are numerous ways in which ML is used in robotics, ranging from autonomous navigation, object recognition, predictive maintenance, and human–robot interaction to motion planning, grabbing and manipulation, anomaly detection, quality control, energy optimization, and reinforcement learning (Standard, Bots, 2025).
- Big data: The big chunk of information harnessed through sensor technologies or relevant recording equipment in farming operations (DLL Group, 2025). Big data, in concert with other technologies such as IoT devices for data collection, have enormous potential to improve profitability and the quality of agricultural products. Future predictions suggest that these data could be stored and accessed through blockchain applications. Such information can be retrieved from anywhere at any time, allowing farmers to make informed decisions via devices such as smartphones, without needing to be physically present in the farm offices. Big data serve as the strategic backbone of agricultural robotics, transforming raw sensor inputs into actionable intelligence and enabling smarter, faster, and more sustainable farming operations (Zaborowicz and Frankowski, 2025).
- Sensors: A recent article published in Agronomy comprehensively explored the integration of AI, sensors, and robotics in modern agriculture, highlighting their potential to change farming practices and drive sustainable development (Pal and Joshi, 2023).

Agricultural robots utilize various sensors to perceive and interact with their environment, including RGB cameras, vision sensors, GNSS, and tactile sensors, enabling tasks such as crop monitoring, soil analysis, and navigation. At the core of the current smart farming revolution are sensor technologies that provide data-driven insights and enable precision PA techniques. These sensor technologies enable the monitoring, measurement, and management of the farming ecosystem in increasingly complex ways. Plainly, in the absence of these technologies, we are unlikely to address the agricultural challenges of the future (Sharp, 2025). The integration of AI, robotics, and sensors in smart agriculture has several key implications. Precision farming utilizes these technologies to collect and analyze data on soil, water, crops, pests, diseases, weather, and market conditions. It provides tailored recommendations for fertilization, irrigation, pest control, and harvesting, optimizing practices for individual fields, crops, and farmers. Autonomous machinery operates with minimal human intervention, performing seeding, weeding, spraying, and harvesting tasks. This reduces labor costs and risks while enhancing efficiency and accuracy. Monitoring of crops is achieved through the use of AI, sensors, and robotics for tracking growth, health, and quality in conjunction with the use of drones, satellites, cameras, and smartphones. It detects diseases, pests, and stresses and offers real-time feedback to farmers. Additionally, AI, sensors, and robotics enable pest and disease detection by identifying and classifying crop threats through drones, cameras, and smartphones. Early warnings and prevention measures are provided, enhancing crop protection. Yield prediction estimates crop yield and quality, aiding in planning, management, and marketing (Pal and Joshi, 2023).

- Global Positioning System (GPS): For outdoor navigation, robots use the GPS, which uses satellite signals to pinpoint their location and plot their routes. The robot’s GPS receiver measures the time it takes for signals from several satellites to arrive in order to determine its latitude, longitude, and altitude. In situations where GPS signals may be poor or sporadic, these data are supplemented with onboard sensors (such as wheel encoders or inertial measurement units) to improve location estimation. For instance, delivery robots in cities use GPS to follow predetermined routes, while agricultural robots rely on it to navigate pre-mapped routes for planting or harvesting (Milvus, 2025).

## 5 How agricultural robotics contributes to sustainability

**FIGURE 5:**
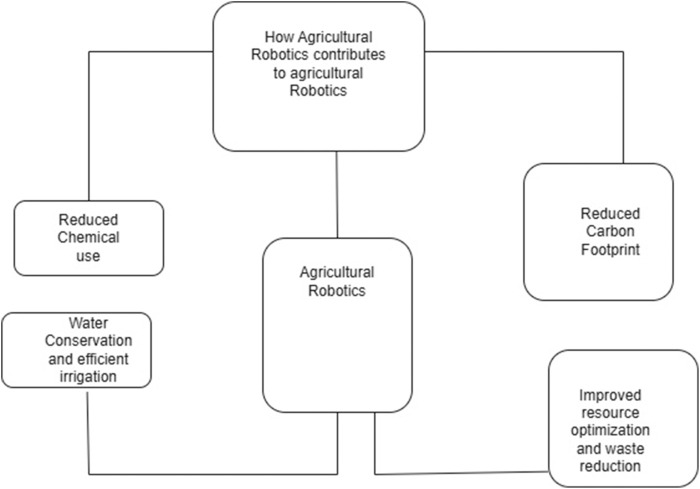
Agricultural robotic impact on sustainability.

Global agriculture is increasingly under pressure to produce more food with dwindling resources, making sustainability a defining priority. Agricultural robotics offers a transformative solution by integrating intelligent automation into farming practices. These technologies, from autonomous tractors and drones to sensor-equipped robots, support multiple dimensions of sustainability by enabling precise management of resources, environmental impact reduction, and improved efficiency of operation. By minimizing chemical input, optimizing water use, and reducing greenhouse gas emissions, robotics contributes directly to environmental sustainability. Economically, robotic systems help reduce labor costs and boost productivity, while socially, they address rural labor shortages and empower smallholder farmers through accessible, scalable tools. This section examines how agricultural robotics is becoming a cornerstone of sustainable development, reshaping the future of agriculture through innovation, resilience, and responsible stewardship of natural resources, as shown in Figure 5.

### 5.1 Environmental sustainability

**FIGURE 6:**
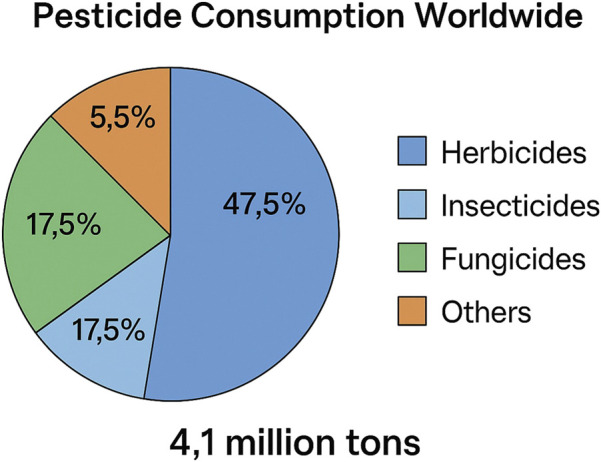
Pesticide.

1. Minimization of chemical use: In general, the use of chemicals is vital in modern agriculture as it facilitates the control of weeds and pests, subsequently ensuring sustainable crop productivity (Parven et al., 2025). Spraying is used in several applications, including herbicides to reduce weeds competing with crops and fungicides to control fungal infections. Furthermore, insecticides are applied to manage insect pests, and micronutrients, such as manganese and boron, are sprayed to supplement essential nutrients (Lochan et al., 2024). Practically, an estimated 4.10 million tons of pesticides are consumed worldwide, with herbicides constituting 47.50%, followed by insecticides at 29.50%, fungicides at 17,50% and others at 5.5%, as shown in Figure 6.

Using precision farming technologies for crop protection, environmental sustainability is promoted through a reduction in pesticide, fungicide, and herbicide use (Saiz-Rubio and Rovira-Más, 2020; Obeng-Ofori et al., 2025). This reduction is achieved with ultra-precise smart spot spray technology, which applies products only where required, thereby minimizing chemical inputs (Anne et al., 2024). Evidence of the impact of this approach is provided by Anastasiou et al. (2023), where precision farming technologies achieved up to 87% savings on herbicides, reduced the area requiring insecticide application by approximately 70%, and decreased weed density by roughly 89%. Further evidence of targeted spray technology in agricultural robotics is presented by the Association of Equipment Manufacturers (2021), which asserts that precision in pesticide application has the potential to reduce usage by approximately 90%.

1. Water preservation and effective irrigation.

**FIGURE 7:**
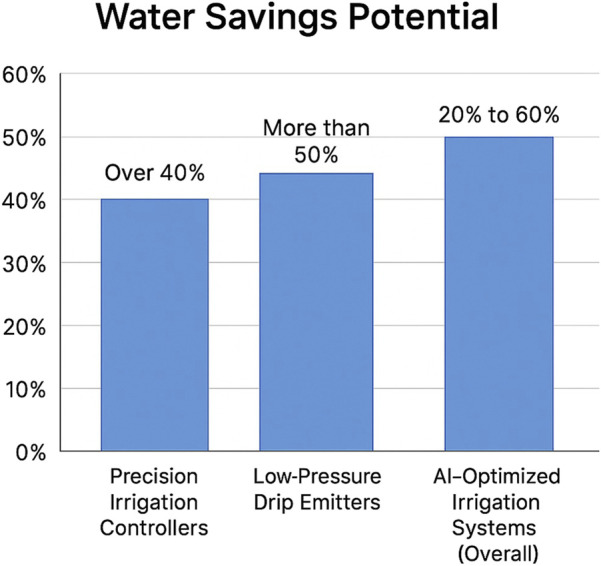
Water-saving potential.

Robotic irrigation systems significantly improve water efficiency by optimizing water use based on real-time data (Pandao et al., 2021). Recent studies reveal that globally, 85% of fresh water is used for agriculture, and current methods for crop maintenance can be improved. Despite being in its infancy, robotics contributes to SDG 6 (Ensure availability and sustainable management of water and sanitation) through inspection and maintenance robots that help preserve water resources (International Federation of Robotics, 2024). Through robot deployment, it is feasible to conserve more water by enabling co-workers to interact with irrigation technology at the level of individual plants (Anderson, 2016). Robotic systems and drones equipped with moisture sensors collect data on crop water requirements, allowing mobile robots to adjust irrigation systems and deliver water precisely where it is needed (Hernández et al., 2025). The COALA project in Australia has demonstrated a 20% increase in irrigation efficiency. This efficiency brings with it the benefits of increased crop yields of between 20% and 30% (Abramov, 2024b). Furthermore, other studies reveal 20%–60% less water with the best systems capable of lowering water use by 40% (Abramov, 2024b). Simply put, there is precision in water distribution, meaning that water is available where it is needed (Hernández et al., 2025). Figure 7 depicts the water-saving potential of robotic technologies. AI-optimized robots appear to have better potential than other technologies.

1. Enhanced soil health and reduced compaction: Soil health and compaction are crucial for farmers and sustainable food production. In general, soil compaction is considered one of the major soil degradation challenges and a fundamental factor in global crop productivity (Zhang et al., 2024). In practice, soil compaction alters soil density and reduces porosity, negatively affecting crop yield and damaging the environment (Roberts-Elliott et al., 2022). Soil compaction has become a major issue in crop farming in recent years. Soil preservation is an increasingly important global topic. Yield variability is influenced by soil management and can range from 10% to 15%.
2. Reduced carbon footprint: Agricultural production has long relied on fuel-powered machines that are usually associated with greenhouse emissions. Agricultural carbon emissions are a fundamental component of global emissions (Nsabiyeze et al., 2024). Minimizing carbon emissions in the agricultural sector is crucial to ensure food security and human prosperity (Yang et al., 2023; Vahdanjoo et al., 2023). However, according to the Food and Agriculture Organization of the United Nations (2024), on a global scale, agrifood systems are responsible for nearly one-third of the total anthropogenic greenhouse gas emissions. The sources of these emissions originate within the farm gate and include livestock and crop production, deforestation, land-use change, and biomass fires. In pre- and post-production processes that comprise the supply chain, which include the manufacture of food, retail, consumption in households, and food disposal, peatland degradation processes are frequently associated with land clearance for agriculture.

**FIGURE 8:**
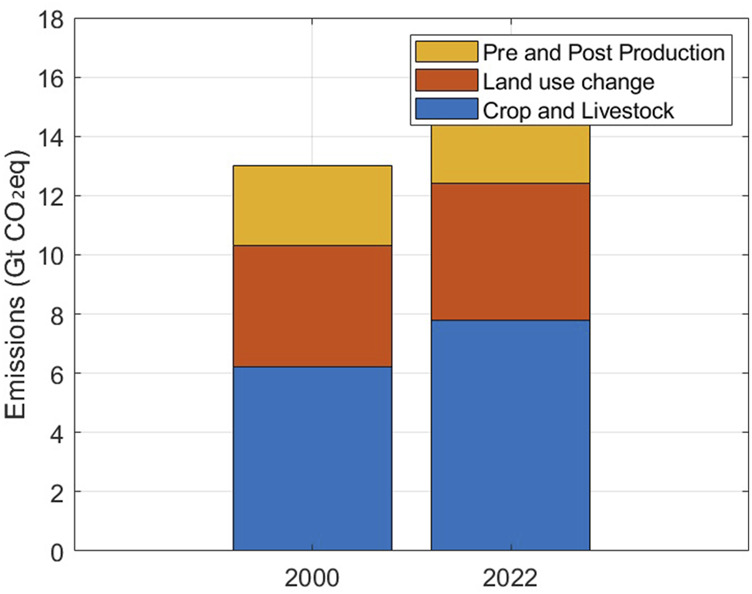
Carbon emissions.

In place of conventional diesel-powered equipment, autonomous electric agricultural machinery and robots can reduce carbon emissions (AgTech Digest, 2025), and emissions can be reduced by improving operational efficiency and reducing fossil fuel dependence (Nagaraja et al., 2024). A case in point is that of robotic tractors equipped with hybrid energy systems, which have the potential to control pests and weeds while reducing emissions by up to 50%, thereby limiting levels of carbon monoxide and nitrogen oxides, as shown in Figure 8. Another positive statistic is provided by China, where agricultural carbon emissions have exhibited a notable declining trend alongside increasing levels of agricultural innovation and technological diversity (Tang et al., 2025).

### 5.2 Economic sustainability

1. Cost savings and resource optimization: Recent trends in multifunctional robots that perform tasks such as planting, fertilizing, and weeding are a positive step toward sustainable food production. These multifunctional robots are versatile machines that consolidate numerous tasks into one, eliminating the need for separate equipment and reducing operational costs. As a consequence, these multifunctional robots streamline processes simultaneously, enhancing production and offering a cost effective solution, subsequently adding value for large-scale operations (Folio3 AgTech, 2024). Furthermore, the integration of AI, ML, and analytics in agricultural robotics results in the development of intelligent and complex farming systems (Agritecture, 2025). This has translated to advancements in real-time analytics, predictive maintenance, and optimization of resources (Elufioye et al., 2024). A combination of AI and IoT technologies maximizes resource use, enhances production control, and lessens reliance on labor. The role of the IoT extends beyond simple automation; it includes improvements to the agricultural supply chain, including decision support systems, predictive analytics, and precision farming. Subsequently, this also results in increased yield and quality. For more detailed information, we refer the interested reader to Naim et al. (2025) and Farmonaut (2025).

### 5.3 Social sustainability

1. Addressing labor shortages and improving conditions: Agriculture is a labor-intensive sector (Martin et al., 2022). Individuals are prone to back injuries when performing tedious and repetitive tasks. In some instances, workers are exposed when handling pesticides, fungicides, and herbicides. It is evident that robots and automated machinery are well suited for labor-intensive tasks that require workers to spend long hours in the fields. Robots can therefore operate in rough and hazardous conditions without fatigue or risk of injured. This will enable farmers to save money and provide a reliable and consistent source of labor (Teal Communications Staff, 2023). In the context of social sustainability, agricultural robots increase productivity while also contributing to SDG 8 (Decent Work and Economic Growth). Statistics reveal that 50% of farm costs are attributed to labor, and 55% of farmers are affected by labor shortages, subsequently shifting approximately 31% of farming activities to less labor-intensive crops (Timoshenko, 2023; Plug and Play Tech Center, 2025). By reducing the cost of labor, robots support farmers’ livelihoods while simultaneously generating new job opportunities. A concrete example is provided in Plug and Play Tech Center (2025), wherein one strawberry robotic harvester has the capacity to harvest a 25-acre area within 3 days, ultimately replacing 30 farm workers.

Beyond labor substitution, agricultural robotics is increasingly associated with measurable job creation and rural development across multiple regions. We examine the job creation landscape through the lens of both developed and emerging economies. For illustrative purposes, Australia and the United States represent the developed economy perspective, while China, Brazil, and India serve as examples from the emerging economy context. In Australia, SwarmFarm Robotics has created more than 60 high skilled jobs in regional Queensland and deployed over 135 autonomous robots operating across 6 million acres, stimulating local engineering and field support services (Queensland, 2025). In the United States, the robotics market was worth approximately USD 14.7 billion in 2024. The market supported approximately 18,000 direct rural jobs in precision agriculture services, robotic maintenance, and data analytics, with the USDA reporting a 24% annual increase in tech-enabled agricultural occupations (Today, 2025). China’s rural revitalization policy has resulted in more than 500,000 rural residents being trained in digital agriculture and the creation of over 200,000 new agritech positions, particularly in UAV operation and smart equipment management. In Brazil, the introduction of Solix Ag’s solar-powered robots has led to a 30% expansion of rural service cooperatives offering robotic weeding, sensor-based mapping, and agro data analysis. In India, government-supported drone training programs have certified more than 12,000 rural drone pilots, contributing to the rapid growth of agritech enterprises and the creation of an estimated 100,000 new jobs anticipated by 2030.

1. Improved accessibility and food security: Although sufficient food is produced today to feed everyone on the planet, access is a challenging aspect. Approximately 80% of the world’s extreme poor living in rural and remote areas are the most affected by natural and man-made disasters, and it is a struggle for them to gain access to training, finance, and technology (Russell, 2022). These affect the food security dimensions, i.e., accessibility, availability, and utilization (Adepoju et al., 2022). For people who are physically unable to obtain food because of physical limitations or other constraints such as age or location, robotics can also be used to improve accessibility. They can benefit from assistive robot arms. Cook meals on their own, but robotic vision systems can assist in identifying ingredients so they are aware of what is available and how to prepare it. Additionally, when conventional modes of transportation are either too expensive or unavailable for them to use independently, robotic delivery services can deliver meals right to their doorstep (Smriti, 2023).
2. Promoting economic expansion and rural development: Maximizing yields per acre is the main objective of integrating robotics into agriculture in order to boost productivity. This is accomplished by using precise farming methods that improve operational efficiency by integrating automation and data-inspired decision making. Precision robotics, for instance, is used by businesses such as Blue River Technology to distinguish crops from weeds and apply treatment selectively, improving both crop yields and quality of produce (Pravin, 2024). According to APET, the use of robotics technology, particularly robotics based mechanization, in African agriculture presents a promising approach to addressing issues related to physical damage, microbial contamination, drought, and heavy rainfall, all of which contribute to the world’s staggering 1.3 billion tons of food waste annually. This is because robots are equipped with sophisticated sensors and algorithms that enable them to precisely harvest crops, determine ripeness, and maneuver through challenging weather conditions. This improves food security and farmers’ livelihoods by lowering food losses and increasing overall crop yields (AUDA-NEPAD, 2023). Due to labor issues, farmers are unable to harvest some crops, resulting in wastage of up to 34% of produce before it even leaves the field. This gap could be addressed by robot solutions, which further reduce food loss and waste, which are significant sources of greenhouse gas emissions (Cleantech Group, 2023). Moreover, after being harvested on farms and before it reaches the retail stage, more than 13% of food is currently lost worldwide in the supply chain. Additionally, according to UNEP statistics, 19 percent of food waste occurs in retail, food services, and at household level (Cleantech Group, 2023; HowToRobot Editorial Team, 2024).

Table 1 outlines baseline studies used for counterfactual analysis in agricultural robotics, comparing outcomes such as yield, water use, emissions, and soil health against conventional practices. Its implication is that robotics can be empirically evaluated for sustainability impact, enabling evidence-based policy and investment decisions tailored to specific environmental and economic metrics.

**TABLE 1:** Baseline studies for counterfactual analysis in agricultural robotics and sustainability.

Outcome	Baseline (counterfactual)	Notes/application
Yield (kg/ha)	Shrivastava et al. (2022)	While it does not quantify yield improvements in kg/ha, it provides a foundational overview of how robotics enhance productivity through targeted interventions such as spot irrigation, crop monitoring, and labor substitution
Water (L/ha)	Dechemi et al. (2023)	Includes case studies where robotic irrigation systems reduced water usage by up to 2,500 L/ha compared to conventional methods
Emission (kgCO_2_e/ha)	Li and Chen, (2024)	Empirically verifies an inverted U-shaped link between robotics adoption and agricultural carbon emissions across Chinese provinces. Emissions are quantified in kgCO_2_e/ha, with regional variation
Energy use (MJ/ha)	Singh et al. (2024b)	Cites energy consumption benchmarks in MJ/ha for various robotic platforms and compares them to conventional mechanization
Economic sustainability	Spagnuolo et al. (2025)	Includes comparative cost analysis of robotic vs. conventional systems, with emphasis on lifecycle costs and productivity gains
Soil health/Chemical input use	Nayak et al. (2022)	Includes data on reduced chemical input per hectare and improved soil structure due to fewer field passes. Provides a lifecycle perspective on robotics and their environmental footprint, useful for counterfactual analysis against manual or hybrid systems

## 6 Case studies and comparative insights

Increasingly, population growth, climate change, and resource scarcity are gaining traction, subsequently increasing pressure on the agricultural sector. To this end, the agricultural industry is expected to increase food production using limited resources that have less environmental impact. Developments in agricultural robotics have demonstrated significant potential across numerous agroecological and economic scenarios, wherein innovative solutions have been developed to address local needs within existing constraints in both developed and emerging economies. In developed economies such as the United States, Australia, and Europe, these innovative robotic solutions have fueled increased farm automation and optimized efficiency in planting, weeding, harvesting, livestock production, and distribution. In emerging economies, agricultural robotics is drifting toward accessibility and sustainability, primarily empowering smallholder farmers, optimizing resources, and promoting ecological resilience. Thus, in this section, we explore the case studies from both developed and emerging economies.

### 6.1 Developed economies

1. United States of America: In 2023, approximately 86.5% of U.S. households had food security the entire year, consistently having access to food for a healthy and active life for all members. The remaining 13.5% experienced food insecurity at some point during the course of the year; this is a statistically significant improvement from 12.8% in 2022. Among these, a subset faced very low food security, the most severe category, where members experienced reduced food intake and altered eating patterns due to limited financial or other resources. In 2023, 5.1% of households fell into this category, a figure that remains statistically unchanged from the previous year (Rabbitt et al., 2024). Furthermore, the same report presents the statistics in Table 2.

**TABLE 2:** Key indicators of food insecurity in the United States as of 2023.

Indicator	Statistic	Source
Food insecure households	13.5% (18 million households)	USDA economic research service
Individuals affected	47.4 million people	Feeding America
Children in food insecure homes	13.8 million	Feed America report
Very low food security	5.1% of households (6.8 million)	USDA ERS
Single mother households	34.7% reported food insecurity	FRAC report
Racial disparities	Black: 23.3%, Latino: 21.9%, white non-Latino: 9.9%	FRAC report

As the world’s largest robotics user with a broad adoption across sectors, the United States of America (U.S.) has integrated robotics in milking systems, autonomous tractors, and unmanned aerial vehicles for monitoring and spraying crops. In 2024, North America accounted for 36.4% of the global agricultural robotics market, with a value of USD 14.74 billion (Grand View Research, 2024). With regard to sustainability practices and environmental impact, the adoption of robotics in the U.S. enables sustainable farming practices through precision input application, leading to the reduction of chemical use and fuel consumption (Grand View Research, 2024; Hoque and Padhiary, 2024). Integrating robotics with data analytics and precision agriculture improves yields and quality of produce while also reducing labor costs. However, real-time robotic operations require an effective communication infrastructure, which is still a major barrier in the U.S. (Zhivkov and Sklar, 2022). Other barriers to widespread adoption include high setup costs, the need for skilled operators, and challenges integrating robotics with diverse farm management systems. Given the heterogeneity of U.S. agriculture, adaptable and flexible robotic solutions are essential. To overcome these challenges, continued innovation is critical. Current efforts focus on improving communication systems, enhancing decision-making algorithms, and developing multi-robot coordination platforms, positioning the U.S. to maintain its global leadership in agricultural robotics.

1. Australia: Australia is a major representative of the developed economies and has made strides in both adoption and deployment, as depicted in Table 3.

**TABLE 3:** Robotic impact on sustainability in Australian agriculture.

Sustainability pillar	Robotic impact	Examples and notes
Environmental	Precision spraying reduces chemical use, autonomous machines lower fuel consumption, and soil health is preserved via minimal tillage	SwarmFarm robotics’ SwarmBots used for efficient crop monitoring in Western Australia
Economic	-Cuts labor costs amid shortages, boosts yields through better decisions, and enables scalable operations for large farms	Orion auto harvester improves productivity and lowers labor costs
Social	Reduces physical strain on workers, attracts youth to farming careers, and enhances food security through reliable production	Murdoch university engages students with autonomous farm vehicles such as robotriks RTUv4

In Queensland, Australia, the SwarmFarm robotics company has deployed more than 135 robots, which now improve farming efficiency across over 6 million acres. SwarmFarm’s autonomous robots are designed to carry out PA operations, such as seeding, spraying, weeding, and mowing (Queensland, 2025). To date, progress has included the development of multiple autonomous robot platforms, along with successful deployment in various agricultural applications, such as planting and harvesting. The deployment of SwarmFarm robots has also led to the creation of high-skilled technology jobs in regional Queensland, including 17 staff designing and building robots. In essence, there has been an overall significant reduction in chemical usage and soil compaction on participating farms. Subsequently, these robots promote and achieve environmental stewardship, such as a reduction of up to 80% in chemical use through a precise application. The use of lightweight machinery reduces soil compaction, allowing for more timely and precise operations, which in turn leads to improved crop yields. Finally, the result is reduced labor costs and greater efficiency.

### 6.2 Emerging economies

1. China: China’s agricultural sector is being transformed by robotics (Saputra, 2024). To date, China is rapidly surpassing Japan and Germany in industrial robotics, leading with 470 robots per 10,000 employees in 2023. This figure obscures Japan’s 419 and Germany’s 429 robots, demonstrating China’s aggressive automation drive. Globally, China accounted for 51% of robot installation, while domestic manufacturing comprises 47 percent of the market, an increase from 28% a decade ago. China’s growth is driven by global investments and strategic policies, asserting its role as one of the world’s fastest-growing robotics markets globally (Sgueglia, 30 January 2025). Agricultural robots are increasingly being integrated into animal monitoring and crop planting, watering, harvesting, and crop health monitoring (LinkedIn Editorial, 2025). As shown in Table 4, robotics adoption has observed a 30% improvement in productivity and up to a 40% decrease in labor costs. Government funding amounting to over USD 1 billion has been used to support the adoption of agricultural technologies. The agricultural robotics market in China is projected to expand to approximately four billion by 2027, driven by the demand for automation in large-scale farms. A number of startups are also emerging in the sector, as exemplified by XAG, Jiangsu Lanjiang Intelligent Technology, Maifei Technology, TopXGun, and Rippton (Tracxn Technologies, 2025).

**TABLE 4:** Robotic impact on sustainability in Chinese agriculture.

Sustainability pillar	Robotic impact	Examples and notes
Environmental	Precision weeding and planting reduce chemical runoff and soil degradation. AI-enabled robots optimize irrigation, and fertilizer use reduced carbon footprint via electric and autonomous machinery	Robots in Tinglin township use 5G and image recognition to distinguish weeds from crops, operating 8 h on a 1-h charge^[1]^
Economic	Boosts total factor productivity (TFP) through automation, reduces reliance on manual labor, and enhances efficiency in high-output regions	Industrial robots significantly increased TFP in 286 Chinese cities, especially in eastern megacities^[2]^
Social	Addresses rural labor shortages, supports aging farming populations with assistive technologies, and promotes digital literacy and tech-based employment	Pilot programs in rural Japan and Kenya show improved retention of youth in agriculture through AI-enabled tools and training platforms

In the animal husbandry domain, a herding robot has been developed in Shanghai (Xinhua News Agency, 2024).

Brazil is among the leading agricultural producers in the world, excelling in the cultivation of soybeans, sugarcane, coffee, and beef production.

**FIGURE 9:**
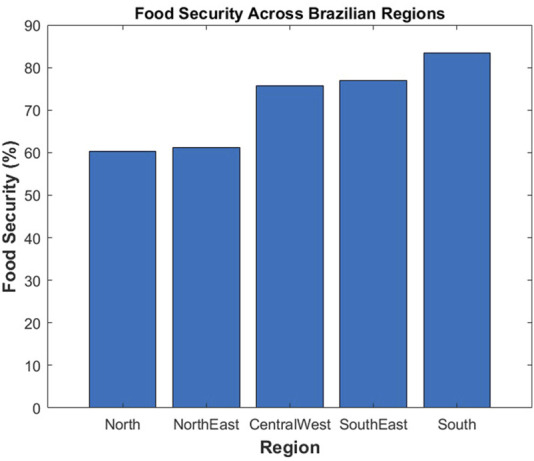
Food-secure regions in Brazil (Agency, 2024).

According to Figure 9, which illustrates the Brazilian food security landscape, the south (83.4%), southeast (77.0%), and central-west (75.7%) regions report the highest proportions of food-secure households, while the north (60.3%) and northeast (61.2%) show significantly lower levels. Despite being a global agricultural powerhouse, nearly 30.7% of Brazilians experienced moderate or severe food insecurity in 2022. Rural areas are especially vulnerable, with food insecurity rates 1.2 times higher than urban zones (Bank, 2023). Brazil uses state-of-the-art robots in agricultural production (Cultivar, 2024). The most common robots are for the identification of weeds, pest infestation, and potential yield (Cultivar, 2024). Machines designed for harvesting grains, fruits, and vegetables are becoming increasingly widespread. According to a study by the Institute for Applied Economic Research (IPEA), automated harvesting could increase soybean yields by up to 20%. Beyond boosting productivity, this technology also improves precision, reduces operational costs, minimizes losses, and enhances the overall quality of the final product (Pisciotto and Simão, 2024).

Undeniably, the influence of agricultural robotics has enabled new levels of sustainability, which are crucial for the future of this sector, as shown in Table 5. Reducing chemical use, minimizing waste of natural resources, and optimizing labor time are among the benefits of this technology. Sustainability in agriculture also means tailoring technologies to Brazil’s unique environmental conditions. With challenges such as climate variability and the urgent need for more efficient resource management, robotics offers a promising solution. These systems can be customized to address the specific demands of Brazilian farming. One such innovation is the Solix Ag Robotics platform, designed to support rural producers and promote environmental stewardship by delivering real-time, uninterrupted data (Fonseca, 2024). This is the first autonomous, electric, and solar-powered robot for large-scale food production, covering areas of up to 200 ha. It performs localized spraying, controls weeds at an early stage, prevents pests without chemicals, saves herbicides by up to 95%, reduces the carbon footprint in agricultural production, and allows low-impact, high-yield agriculture. The innovation was launched for the production of sugarcane and is also currently used in maize, soybean, wheat, and cotton, among others. Limitations: Climate change and labor shortages threaten crop yields and food security. However, despite its natural advantages, Brazil is facing serious challenges. Along with climate change and labor shortages, it is also encountering environmental pressures, changing consumer demands, and demand volatility. A particular challenge for Brazil is the deforestation of the Amazon, which attracts negative global attention. Together, these factors pose a threat to Brazil’s sustained growth.

1. India: Over 55% of the population relies on farming for a living; India is a major player in the agricultural sector globally. It has the world’s largest herd of cattle (buffaloes), milk and spices production, and the largest cultivated area of rice, wheat, and cotton. In addition, vegetables, fruits, farmed fish, tea, sugarcane, wheat, sugar, and rice are among its leading products (India Brand Equity Foundation, 2025). The growing population has made food security a matter of utmost concern. India will need significant work to address this issue in order to meet the growing food demand, meet sustainable agricultural production, reduce food loss and waste, and guarantee that those who are hungry or malnourished have access to wholesome food. Adopting comprehensive and integrated sustainable farming methods that cover both production and consumption is essential to achieving this. By 2050, India’s population is projected to reach 1.668 billion, necessitating a substantial increase in food production to feed this rapidly growing population (Singh et al., 2024a). Currently, the food security situation is such that approximately 200 million people in India face malnutrition, pointing to food insecurity. However, there is an inconsistency in the data and the level of food insecurity nationwide due to differences in the methodology employed to determine food insecurity status (McKay et al., 2023).

**TABLE 5:** Robotic impact in Brazil by sustainability pillar.

Sustainability pillar	Robotic impact
Environmental	Reduced chemical inputs, optimized water use, and lower emissions
Economic	Increased productivity (up to 30%), reduced labor costs
Social	Mitigates rural labor gaps, supports smallholder resilience

According to Anonymous (2025), the Government of India has several initiatives to alleviate food insecurity including the following.

- National Food Security Act (NFSA): provides food grains that are subsidized to approximately 67% of the population,
- Public Distribution System (PDS): provides food grain subsidies to the less privileged and the poor.
- Mid-Day Meal Scheme (MDM): A school feeding program that provisions meals to children in both primary and secondary schools. The program aims strives to improve the nutritional status of children while incentivizing school attendance.
- Pradhan Mantri Garib Kalyan Anna Yojana (PMGKAY): This is a COVID-19 relief initiative launched in 2020 by the government. The initiative provisions 5 kg of grains per individual every month to 80 crores people for 8 months.

The demand for increased food production, combined with the adoption of precision farming methods, is driving a notable surge in agricultural robotics in India. With an emphasis on efficiency and minimizing human work, robots are being developed for a variety of jobs, such as planting, weeding, spraying, harvesting, and crop monitoring.

In India, agricultural robotics is developing quickly and providing creative answers to the various problems farmers encounter. While drones and ground-based robots continuously monitor crop health, nutrient levels, and insect infestations, precision seeding and planting robots are enhancing seed placement for higher yields. Herbicides are used less frequently when weeding robots employ AI to accurately target undesirable plants. By applying fertilizers and pesticides more effectively, microspraying robots reduce negative environmental effects. Robotic harvesters and sorting equipment expedite the collection and processing of crops, and automated irrigation systems optimize water usage based on moisture in the soil and meteorological conditions. Multispectral camera equipped drones offer precise yield estimations and crop health evaluations. Advanced navigation systems enable autonomous tractors to carry out duties such as farming and plowing, while robots for livestock management take care of feeding, milking, and health monitoring. While labor-saving tools, such as exoskeletons and robotic arms, assist with precise tasks, post-harvest robots support packing and sorting. Furthermore, environmental monitoring robots support sustainability initiatives by tracking pollution levels from agricultural processes (Manohar and Chaithra, 2024).

### 6.3 Comparative sustainability outcomes

In both developed and emerging economies, agricultural robotics generally presents positive comparative sustainability outcomes compared to traditional large-scale mechanized agriculture, primarily by enabling precision agriculture. The sustainability results for five countries are presented in Table 6.

**TABLE 6:** Comparative sustainability outcomes of agricultural robotics across five countries.

Country	Environmental outcome	Economic outcome	Social outcome
United States	• Reduced chemical and fuel use through precision spraying and autonomous machinery • Improved soil health from minimized compaction	• Lower labor costs in large-scale farming • Higher yields through automation and data-driven operations	• Mitigates labor shortages • Enhances worker safety by reducing exposure to hazardous tasks
China	• Reduced chemical runoff via precision weeding and planting • Lower carbon footprint through electric/autonomous platforms	• Up to 30% productivity increase • Labor costs reduced by ∼ 40%	• Supports rural revitalization • Attracts youth to digital agriculture
Brazil	• Up to 95% herbicide reduction (Solix Ag robot) • Improved soil and water stewardship	• Productivity increased up to 20%–30% • reduced operational costs in soy, maize, and sugarcane production	• Strengthens smallholder resilience • Addresses rural labor gaps
Australia	• Up to 80% reduction in chemical use (SwarmFarm robots) • Reduced soil compaction via lightweight platforms	• Lower labor dependency • Enhanced yields through precise autonomous operations	• Less physical strain for workers • Supports training and engagement of youth in agritech
India	• reduced pesticide and fertilizer misuse through microspraying and precision seeding. • water savings via automated irrigation	• Higher efficiency in planting, weeding, and harvesting • Lower post-harvest losses	• Supports food security • Addresses acute labor shortages in rural farming communities

Robotics provides clear sustainability advantages in both emerging and developed economies. Environmentally, it enables precision agriculture, leading to substantial cuts in resource use: chemical input can be reduced by 20−−95% , water efficiency increases by 20−−60% , and carbon emissions fall due to the adoption of electric platforms. Economically, automation improves sustainability by lowering operational costs and improving overall yield quality. Socially, robotics helps mitigate labor shortages and successfully attracts young people to modern agritech professions.

## 7 Challenges and opportunities in adopting agricultural robotics for sustainability

Agricultural robotics and other related emerging technologies have fundamentally transformed agricultural production systems, leading to greater efficiency and sustainability. The complete adoption and full sustainability of agricultural robots depend on overcoming several key hurdles: high costs, technical shortcomings, interoperability, and ethical concerns (Urrea and Kern, 2025; Nagaraja et al., 2024).

### 7.1 Limitations, uncertainties, and benchmarking against existing innovations

While this review highlights several promising innovations, it is important to recognize the associated limitations and uncertainties. Many of the benefits outlined in Section 6 are derived from controlled experimental settings, and their performance may not translate consistently to real-world farm conditions. However, robotics remain largely untested in the long term, particularly regarding energy demands, sensor deterioration, and e-waste management, and there is also an economic challenge to consider. For example, the ability of robotics to decrease labor demand and improve precision represents a valuable market contribution. However, due to high installation and maintenance costs, adoption varies between large commercial operations and smallholder farms. Undeniably, areas with underdeveloped technical infrastructure will definitely not experience the same performance gains observed in highly digitized agricultural systems. Comparative benchmarking against existing innovations reveals additional differences. Non-robotic precision agriculture tools such as smart irrigation systems, handheld sensors, and improved crop varieties can provide substantial sustainability benefits at a lower cost and complexity. In some contexts, traditional low-tech innovations, such as conservation agriculture, farmer-led integrated pest management, or renewable-energy-based irrigation, outperform robotics in cost-effectiveness and scalability. These alternatives highlight that agricultural robotics should be considered a complementary rather than singular pathway toward sustainable food systems. Finally, while robotics offers transformative potential, its benefits must be interpreted within these economic, environmental, and social constraints. Future evaluations should incorporate long term field data, broader geographic comparisons, and multi-technology benchmarking to provide a more complete sustainability assessment.

### 7.2 Technical and operational challenges

- Interoperability: sustainable food security requires research and innovation to also include the development of robust robotic platforms suited for agricultural environments. Sensing and perception capabilities remain fundamental to effective robotic operation in agricultural environments. Equally important are planning and coordination, manipulation and grasping, learning and adaptation, and interoperability between robots and other farm machinery. Human–robot collaboration is also essential, particularly regarding safety considerations and user acceptance (Duckett et al., 2018). Robot and sensor technologies are increasingly being adopted in several agricultural applications, including land preparation, weed management, harvesting, fertilization, and crop monitoring and analysis. The widespread adoption of smart farming technologies is currently hindered by a lack of interoperability and the prevalence of closed, manufacturer-specific solutions. This forces farmers to make difficult choices regarding devices, sensors, and data platforms, limiting their ability to flexibly adapt systems to their individual needs and restricting knowledge exchange (Milella et al., 2024).

Interoperability: Data serve as a foundational element in the success of smart agricultural systems. It typically originates from a wide range of heterogeneous sources that include thousands of individual farms, livestock facilities, and enterprise applications and exists in diverse formats. This diversity makes bringing together these elements a challenging task. This highlights that data interoperability is crucial for unlocking the full value of this dispersed information through systematic collection, storage, processing, and knowledge extraction. Similarly, effective communication between heterogeneous devices requires seamless interconnection and interoperability. Cross-technology communication frameworks can significantly improve system-wide integration and performance.

- Standardization: To derive benefits from digital technology in smart farming, device standardization is vital. It facilitates compatibility between and across platforms, reduces the risk of misinterpretation, and minimizes inconsistencies in output caused by system modifications over time. With interoperability and standardization, issues of devices, systems, and applications can also be resolved (Abbasi et al., 2022). However, inadequate interoperability can lead to data silos, inefficient task coordination, and increased operational complexity, thus undermining the benefits of automation. The impact of inadequate interoperability is articulated by Vivian (2022) as data silos and fragmentation, delayed decision-making, and limited scalability, along with security and privacy risks. Data silos and fragmentation imply isolated systems where data remain locked within a specific platform or device. A case in point is when smart tractors, irrigation controllers, and crop sensors fail to communicate, leading to farmers missing out on comprehensive insights that could guide better decision-making. Furthermore, inadequate interoperability increases costs and brings redundancy. Costs increase when farmers have to invest in multiple devices and software platforms that perform similar tasks but cannot be integrated. This results in redundant infrastructure and increased operational costs. A case in point is that the existence of separate cloud dashboards for weather data, soil moisture, and drone imaging can be both inefficient and expensive. Another impact pertains to delayed decision-making, where inadequate interoperability results in delays in data transmission and analysis. A concrete example of such a scenario is in precision agriculture when a pest detection sensor is unable to integrate with a crop management system. For such a scenario, an immediate action such as spraying pesticides can be delayed, subsequently leading to crop damage. In other words, there is limited scalability of systems as inadequate interoperability hinders plug-and-play scalability. The key takeaway is that inadequate interoperability also introduces security and privacy risks as farmers are forced to use multiple isolated platforms with inconsistent security protocols, creating opportunities for data breaches and unauthorized access.

Overall, achieving standard communication protocols and modular architectures will be absolutely important for scalable, cross-platform robotic ecosystems.

- Robustness: it determines how well robotic systems perform when faced with changing field conditions, uneven terrain, variable weather, and biological variability in crops. Agricultural robots must be capable of navigating through variable farm layouts and around outdoor obstacles. This will involve, for example, navigation through swamps, logs, pits, and uneven terrains. A robust navigation system is, therefore, a fundamental requirement for these robots to operate effectively in a complex outdoor environment and make accurate topological assessments (Hokuyo USA, 2025b). Inadequate robustness brings to the fore issues such as operational failure and downtime, high maintenance and ownership costs, reduced adoption and farmer trust, data quality problems and decision errors as well as limitations in scalability and contextual fit. From the operational failures and downtime perspective, the primary cause of operational failures and downtime stems from the harsh operating environment of the farm. Practically, agricultural robots persistently face environmental challenges such as dust, moisture, uneven terrain, and temperature extremes. Certainly, when robot design incorporates inadequate robustness, these environmental challenges quickly overwhelm the system. This insufficient resilience leads to frequent breakdowns, particularly when robots are engaged in time-sensitive and critical tasks such as harvesting or spraying. As a result, this cycle of environmental stress leading to equipment failure directly disrupts farm schedules and dramatically reduces overall productivity and economic returns. In essence, the environment exploits the lack of robustness, causing the mechanical and electronic failures that result in costly downtime (Cheng et al., 2023). On the other hand, high maintenance and ownership costs are associated with the lack of robustness. These are emanating from fragile robotic systems requiring frequent repairs and specialized parts. This challenge is exacerbated in rural and remote areas due to a lack of technical support, thereby increasing downtime and costs. Consequently, smallholder farmers will most likely struggle with affordability and sustainability (Beldek et al., 2025). Additionally, reduced adoption and diminished farmer trust are also consequences of a lack of robustness. Continued system failures directly lead to a crisis of confidence: farmers lose belief in automation when robots consistently fail to perform under real world conditions. When negative and costly experiences occur, these accounts quickly spread through word of mouth, creating skepticism that dramatically slows the regional uptake of new technology. This issue is compounded because trust is particularly fragile in developing regions where farmers often operate with limited financial safety nets and inadequate maintenance infrastructure (Beldek et al., 2025). The issue of data quality and decision errors fundamentally undermines the goals of precision agriculture. Sensor instability, often resulting from insufficient robotic robustness, leads to the collection of inaccurate data. Furthermore, poor robustness negatively affects the seamless integration of robots with IoT and cloud platforms. The combined result is data unreliability that sabotages the accuracy of AI-driven decision support and the effectiveness of precision agriculture strategies.

Beyond data integrity, the challenge of scalability and contextual fit restricts market penetration. Current robotic designs, which are often optimized for large, uniform corporate farms, frequently fail in fragmented or mixed-use plots common across many global regions. This limited adaptability to local variables—such as specific crops, soil types, and climate zones—severely restricts the ability of the technology to scale across diverse agroecological regions, preventing its widespread impact.

### 7.3 Economic and institutional challenges

Economic challenges (Nagaraja et al., 2024) include high initial costs, which are a major impediment to the adoption of agricultural robots (Pandya, 2025). According to Sheykin (2025) the start up costs are a bit prohibitive with the total costs reaching approximately USD974,00. Another economic consideration is the return on investment, which typically takes longer for small- to medium-sized farmers. Essentially, substantial initial costs require significant gains in efficiency and productivity to be justified, and these benefits may not be immediately apparent. Agriculture’s diversity in scale (scale of farms), systems, and environments complicates efforts to define problems with sufficient scope and market relevance to justify targeted innovation or investment. Evidently, the lack of standardization means that a robot designed for a large corn farm in the U.S. may be completely unsuitable for a small, diversified vegetable farm in Europe or Asia (Lemay and Boggs, 2024). Another economic consideration is the return on investment, which typically takes longer for small- to medium-sized farmers. Essentially, substantial initial costs require significant gains in efficiency and productivity to be justified, and these benefits may not be immediately apparent. Agriculture’s diversity in scale (scale of farms), systems, and environments complicates efforts to define problems with sufficient scope and market relevance to justify targeted innovation or investment. Evidently, the lack of standardization means that a robot designed for a large corn farm in the U.S. may be completely unsuitable for a small, diversified vegetable farm in Europe or Asia (Lemay and Boggs, 2024). Given agricultural diversity, robots are mostly designed to automate specific tasks. These tasks range from seeding and weeding to ultra-low-volume spraying. Unlike humans, who are able to switch between tasks, purchasing robots with poor adaptability is high risk (Spagnuolo et al., 2025). As a result, this forces developers and designers to create niche, highly specialized machines. Subsequently, fragmentation of the product market prevents manufacturers from achieving the economies of scale necessary to reduce unit costs, directly contributing to high prices (Lowenberg-DeBoer, 2022).

### 7.4 Environmental and lifecycle impacts

The responsible deployment and long term viability of agricultural robots are contingent upon mitigating their significant environmental and lifecycle impacts, which encompass challenges such as substantial energy use, material waste generation, and potential ecological disruption.

#### 7.4.1 Technical and operational challenges

- Energy consumption: Agricultural robots are increasingly being deployed to optimize energy consumption in crop management. By precisely controlling planting, weeding, and harvesting tasks, robots are able to minimize operation time and power required for machinery, leading to significant reductions in the farm’s carbon footprint. For example, robotic weeding systems can apply energy only where needed, adjusting usage in real time based on weed detection rather than blanket coverage (Hoque et al., 2025; T-Robotics, 2023).
- Durability and maintenance: Hazardous field conditions, such as dust, moisture, and uneven terrain, can accelerate wear and tear on agricultural robots, leading to frequent replacements and increased waste. To address these challenges and extend operational lifespan, lithium agriculture battery packs offer a robust solution. These batteries require minimal maintenance, deliver greater longevity, and exhibit enhanced durability. Engineered to withstand environmental stress and vibrations common in agricultural settings, they can also be discharged to low capacity without significant performance loss. This ensures that autonomous systems remain reliably operational throughout the day (Battery, 2023). Lithium batteries are becoming increasingly economically favorable compared to diesel fuel. The price of lithium batteries per kilogram has steadily decreased, contrasting sharply with the upward and volatile trend of diesel fuel costs due to various contingent factors. Despite this economic advantage, improving the performance of battery-powered systems faces ongoing engineering challenges, specifically the need to reduce the weight and size of the batteries while simultaneously increasing their operating autonomy (Spagnuolo et al., 2025).

#### 7.4.2 Environmental impacts

- Soil compaction: After tillage, traffic compaction is a major disruption to the soil’s structure. A single compaction event may result in alteration of the pore space, negatively affecting pore functionality by preventing gas and water transport, increasing root impedance, and impacting macrofauna and microorganisms (Calleja-Huerta et al., 2024). Lightweight autonomous field vehicles are an alternative to manned vehicles with high wheel loads; their use can lead to a high risk of soil compaction (Calleja-Huerta et al., 2024).
- Chemical overuse: A heavy dependence on agricultural chemicals contributes to the decline of biodiversity, along with a series of social and environmental problems such as greenhouse gas emissions and eutrophication (IDTechEx, 2024). Resultantly, the toxic effects of a growing number of chemical pesticides on humans and the environment have led to a ban on chemicals in most countries, led by the Pesticide Action Network (PAN) (Global Agriculture, 2025).

Countries such as Australia have implemented a ban on certain pesticides, but this solution has led to the overuse of others.

- Habitat disruption: Agricultural robots facilitate reduced habitat disruption by enabling precision practices. However, to ensure environmental benefits, their deployment must be strictly managed for sustainability. Without this oversight, the infrastructure required for large-scale, robot compatible agriculture could indirectly lead to increased habitat loss and decreased biodiversity (Sparrow and Howard, 2021). Autonomous machines operating continuously may disturb wildlife habitats, especially in mixed-use or edge ecosystems.

#### 7.4.3 Lifecycle impacts

- Manufacturing footprint: Manufacturing specialized robotic components, such as sensors and processors, requires the mining of rare earth metals and high energy inputs, leading directly to environmental degradation (Spagnuolo et al., 2025).
- End-of-life disposal: The production and disposal of robotic systems may have environmental consequences (Obeng-Ofori et al., 2025). A major challenge is that none of the robotic components are biodegradable or recyclable, leading to the accumulation of electronic waste if not properly managed (Sparrow and Howard, 2021).
- Software obsolescence: Rapid advancements in AI and robotics technology can render current systems obsolete quickly. Keeping up with technological changes and ensuring ongoing relevance can be challenging. Rapid tech evolution can render robot software outdated, forcing hardware upgrades or replacements sooner than necessary. Invest in upgradeable systems and maintain relationships with technology providers for continuous updates. Stay abreast of technological advancements and plan for regular equipment and software reviews and updates (Portal, 2023).

### 7.5 Cybersecurity and data governance risks

#### 7.5.1 Vulnerabilities in autonomous systems

The widespread adoption of complex, interconnected autonomous robotic systems is increasingly introducing critical cybersecurity risks. Their reliance on integrated hardware and software creates new vulnerabilities that are susceptible to attacks (Maraveas et al., 2024), such as sensor spoofing and adversarial machine learning. At the same time, the massive data streams generated by these systems raise significant data governance challenges, particularly in safeguarding privacy, clarifying ownership rights, and establishing functional interoperability standards to support safe and ethical data exchange. In general, data collection in interconnected robotic systems involves sensor technologies, cameras, and other data collection tools enabling robots to monitor plants and collect data on crop health, soil conditions, and environmental factors (Naim et al., 2025). The massive data stream interchange renders these autonomous systems susceptible to cyberattacks such as sensor spoofing, data injection, adversarial machine learning, remote injection, and remote intrusion. Other system vulnerabilities arise from firmware and software exploitation. In automated vehicles, Global Navigation Satellite System (GNSS) spoofing and injection of fake messages are the most dangerous attacks. GNSS is the key to accurate vehicular positioning on a map. Consequently, manipulation of GNSS data may result in erratic and inaccurate maneuvers, potentially endangering crops and personnel. Hence, it is mandatory for a GNSS signal to be secure. Another form of cyberattack is adversarial machine learning, where deep learning models used for perception and control are vulnerable to adversarial examples—subtle input manipulations that cause misclassifications. These disruptions can lead to failures in object detection and decision-making, which compromise the reliability of autonomous systems (Goodfellow et al., 2014). AML in agricultural robotics refers to the security threat where maliciously crafted input data (known as adversarial examples) are introduced to confuse or manipulate the ML models that control or inform autonomous farm equipment. A third form of attack is remote hijacking and network intrusions (John, 2025). In remote hijacking, drones can be remotely tampered with to return false data or piloted to infiltrate remote Wi-Fi networks (Amoore and Piotukh, 2022). An interesting case is that of the Department of the Interior, which has identified espionage risks associated with its own drone fleets being manufactured by a Chinese company. In 2016, the Federal Bureau of Investigation (FBI) released a joint memo with the USDA warning farmers that they were increasingly at risk of having their data held for ransom or of bulk data theft. In 2017, the U.S. Navy conducted war games designed to train servicemen to protect key sectors, including food and agriculture, against malicious state and extremist actors. Soldiers were trained to mitigate attacks such as remote alteration of temperature readings in a vegetable canning facility and ransomware targeting financial data from an agricultural company. The fourth attack pertains to firmware and software exploits in which unpatched software in embedded systems can be targeted for privilege escalation or system disruption. Thus, software flaws in robotic farming systems pose significant cybersecurity threats that result in severe consequences, such as operational failures, crop loss, financial damage, and even risks to food security and physical safety (Submitted Content, 2025). Furthermore, outdated firmware and software indicate that not all farmers update their equipment on a regular basis, which can result in serious weaknesses.

#### 7.5.2 Data governance vulnerabilities

- Ambiguity in data ownership: Autonomous systems generate vast telemetry and behavioral data, often without clear legal frameworks defining who owns or controls it. The legal landscape surrounding autonomous driving data presents significant challenges, primarily revolving around ownership, data breaches, and liability for infringement (Xu et al., 2024). Undeniably, a number of issues arise, such as who indeed owns the data, increased potential for data breaching, and infringement liability, given the numerous people involved in data handling.
- Privacy and consent challenges: Continuous data collection (e.g., location, biometrics, and environmental) raises concerns about user consent, especially in public or shared spaces (Holm et al., 2024). Two major risks arise that involve reputational damage and unauthorized use of data.
- Cross-border data flows: Global deployment of autonomous platforms (e.g., drones and vehicles) complicates compliance with regional data protection laws such as POPIA, GDPR, or CCPA. However, cross-border data flows also pose challenges among individuals, governments, and businesses as they amplify concerns regarding data protection and privacy, digital security, intellectual property protection, national security, regulatory reach, trade, competition, and industrial policy (Casalini et al., 2022). IoT-based agricultural robots often transmit data across borders using cloud platforms, making them susceptible to man-in-the-middle (MITM) attacks, session hijacking, and remote hijacking of control systems (Adewusi et al., 2022). MITM attacks have adverse consequences for the farming systems, which impact the integrity of transmitted data due to it being altered prior to reaching its intended destination. The inaccurate information further compromises the reliability of smart agriculture systems (Adewusi et al., 2022). On the other hand, hijacked agricultural robots disrupt irrigation, feeding, or harvesting routines, along with causing physical harm to livestock or crops. In some instances, they may trigger ransom demands or data extortion.
- Lack of accountability mechanisms: When autonomous decisions cause harm, it is often unclear whether liability rests with the developer, operator, or AI system itself. However, the design and configuration of intelligent systems lead to harm and unintended consequences in digital agriculture. Invasion of farmers’ privacy, damaging animal welfare due to robotic technologies, and lack of accountability for issues resulting from the use of AI tools are only some examples of ethical challenges in digital agriculture (Dara et al., 2022). For example, if AI systems apply excessive amounts of chemicals or pesticides due to system errors, questions arise regarding responsibility for resulting crop damage. Automated tools, such as harvesting robots, may damage crops during harvesting operations as a result of system errors.

### 7.6 Human–robot interaction and field safety

Human–robot interaction (HRI) in field environments requires extensive training, flexible safety protocols, and user friendly design to lower risks and support teamwork. For this reason, mixed human–robot teams need active risk management strategies that combine technical protections with behavioral awareness.

#### 7.6.1 Human–robot interaction

- Unstructured environments: Agricultural robots are generally expected to operate in unstructured environments wherein there are variable objects. The development of these robots is thus a rather complicated exercise. Agricultural robots must have the capacity to deal with numerous complexities emanating from the dynamic and non-controlled operational environment. In terms of navigation, agricultural robots move on unstructured and unpredictable terrain. In terms of ambient conditions, these robots operate under volatile and uncontrolled climatic environments, such as wet and muddy soil, strong winds, atmospheric dust, and varying light conditions depending on sun position and cloud cover. Furthermore, operations in agricultural tasks are variable and highly complex, being complicated because of the unstructured environment and variability of biological materials (Adamides and Edan, 2023; Nambiar et al., 2025). Without a doubt, robots must adapt to non-uniform layouts, unexpected obstacles, and variable lighting or dust. Currently, robots do not have the capacity to interact well with humans in unstructured human environments. Bringing robotic systems to human environments remains a major challenge (Jägersand et al., 2025).
- Communication and control interfaces: HRI design encompasses physical interaction, user interfaces, and communication to create robotic systems that are easy to control, safe, and productive for human operators. Effective human–robot collaboration in agriculture capitalizes on the combination of robotic precision and endurance with human cognitive skills, enabling systems that are both efficient and socially acceptable. Ongoing studies focus on developing intuitive communication interfaces, improving robot adaptability to diverse agricultural conditions, and embedding safety protocols such as wearable sensors to monitor human activity. This ensures safe and seamless integration into existing workflows. Moreover, HRC systems can be viewed as transitional frameworks that integrate social and technical dimensions (e.g., actively involving human feedback) to enhance acceptance and functionality in real-world settings (AIOTI Working Group on Agriculture, 2025).

Human factors research is needed to improve and simplify display and control interfaces. Two particular problems with human factor implications are (a) provision of 360° observation at the remote site and (b) compensation for intermittent communication delays and dropouts. There are promising developments (Sheridan, 2016). It is easy to observe that many agricultural workers may lack training in complex interfaces. This implies intuitive, multilingual, and limited-bandwidth interfaces are essential for usability in rural settings.

- Trust and transparency: In general, a lack of transparency affects trust, regulatory compliance, and user understanding in high-stakes, safety-critical agricultural tasks (Hamrani et al., 2025). For this reason, farmers may hesitate to depend on robots unless they understand their behavior. Lack of explainability in AI-driven decisions (e.g., why a robot skipped a row) can erode trust.
- Shared autonomy: This is based on achieving an important balance between human oversight and robotic autonomy. In particular, who bears decision-making responsibility when humans and robots interact? More specifically, who is responsible for decision-making in a human–machine relationship? According to Langås et al. (2025), the human autonomy aspect and the existence of frameworks that focus on enhancing societal and psychological industrial impact need consideration. The absence of such frameworks translates to overreliance on automation, leading to complacency and underreliance, which limits efficiency.

#### 7.6.2 Field safety

- Proximity to humans and animals: Modern robotic system design is based on HRC to harness human and robot strengths (Yerebakan and Hu, 2024). A recent practical HRC scenario involves a vineyard spraying robot that was human-assisted (Hadzilacos, 2017). Human activity recognition (HAR) is useful for guiding the robot’s decisions, ensuring that it responds appropriately in collaborative tasks, and operating in a ‘socially aware’ manner. This capability is vital for worker safety whenever humans and robots are occupying the same space. Efficient HRC integration into agriculture requires considering several key factors, ranging from practical concerns such as efficiency gains to critical aspects such as mitigating safety risks. On the other hand, humans and animals are highly dynamic obstacles with unpredictable movements. Animals, in particular, may react violently or panic due to the noise, vibrations, or unfamiliar presence of robots, posing a risk to both themselves and nearby workers.
- Sensor limitations: Modern sensor technology including LiDAR, cameras, ultrasonic, and radar, faces several technical and practical limitations in agricultural settings (Fontani et al., 2025). The challenges emanate from, among others, environmental interference, complex scenarios and camouflage, limited datasets, physical limitations, and the tradeoffs between speed and accuracy. Perception failures may be due to software, sensor hardware, or communication issues. Sensors must conform to defined levels of performance. Hardware sensor properties, such as response time and detection range, can fall short due to issue of design or calibration. Object dimensions, surfaces, environmental factors, positions, and human actions can result in failure. System limitations, sensor interference, and environmental conditions also lead to reduced detection capabilities (Paisley, 2024). Moreover, given complex scenarios and camouflage in modern fields, reliable obstacle detection in agricultural environments is challenging due to complexity and unstructured environments (Hadzilacos, 2017). These obstacles can either be positive or negative. Positive obstacles are denoted by buildings, metallic poles, and trees. The negative obstacles may comprise vehicles, animals, people, ditches, holes, significant slopes, and water. Obstacles may also vary greatly from one situation to another, depending on the type of crop, fruit, vegetable, or plant grown, along with the curvature of the landscape. Currently, robots have limited understanding of such obstacles (Reina et al., 2016). Furthermore, limited datasets have been the order of the day. This means that a lack of large-scale public datasets of diverse agricultural environments and potential obstacles makes it difficult to train robust and highly accurate ML and AI algorithms for perception systems. The effectiveness of robotic safety systems is often constrained by physical limitations, such as insufficient sensor detection range and response time at high speeds, and interference from environmental factors such as smooth or shiny surfaces. Furthermore, the high cost of advanced sensors, coupled with the technically specialized skills needed for operation as well as maintenance, presents a significant financial and operational barrier to adopting HRC technology, particularly for small-scale farming operations (Spagnuolo et al., 2025).
- Cyber physical risks: A general lack of physical security mechanisms renders smart farming equipment vulnerable to cyber-attacks as it can easily be stolen and used to install malware. Malicious actors can exploit weak physical security and tamper with it to install firmware and malware to steal data and control it remotely (Maraveas et al., 2024).
- Emergency response and fail safes: Fail-safe mechanisms are designed with the objective of bringing robots to a safe state in the event of a system failure, unexpected condition, or emergency. Unexpected equipment malfunctions therefore pose a significant safety risk as they can cause automated machinery to operate unsafely or shut down without control, potentially leading to worker injury. Critically, these failures often result in a loss of control of workers, making it difficult to mitigate the problem and increasing the overall danger. In case of an emergency, human workers must have the ability to quickly intervene and stop machines during an emergency (Engineering, 2024).

### 7.7 Equity and inclusion considerations

For smallholder farmers, the sustainable and inclusive adoption of agricultural robots faces important challenges related to equity, inclusion, and affordability that must be addressed.

- Affordability for smallholders: The adoption of robots in sustainable food systems also presents several challenges, such as affordability, skills, and infrastructure (Nxumalo and Chauke, 2025). Numerous digital solutions have been developed to serve the agricultural industry. However, the majority of small-scale farmers cannot access the digital innovations due to either non-existent digital or inadequately developed ecosystems. Among the challenges that render these innovative digital solutions unsuitable for smallholder farming contexts are digital literacy gaps, service costs, limited digital skills, and restricted access to digital technologies. In addition, smart devices and digital infrastructure, such as connectivity and mobile networking infrastructure for the Internet and broadband connectivity, also contribute to the unsuitability of digital solutions (Gumbi et al., 2023). High costs, limited access to technology, and infrastructural deficiencies prevent smallholders from benefiting from AI advancements (Bhat et al., 2025; Food and Agricultural Organization of the United Nations, 2025). Without a doubt, these could benefit from previous Television White Space (TVWS) initiatives (Nleya et al., 2014a; Nleya et al., 2014b; Nleya, 2016) and recent studies such as Chigaba et al. (2025) and Nleya et al. (2025).
- Gendered impacts and access disparities: While agricultural robots have the potential to revolutionize farming, their benefits risk deepening existing gendered access disparities and excluding marginalized smallholders if adoption is not guided by intentional design and policy.
- Local repair and maintenance ecosystems.

### 7.8 Opportunities and pathways forward

- Technical complexity and maintenance: The general lack of specialized technical skills is a major barrier to the adoption of agricultural robots. This is particularly true in rural and remote areas, where most farmers may lack access to essential training or support. In order to achieve this, farmers may need additional training or more skilled personnel to manage these technologies effectively. Moreover, maintaining the optimal performance of agricultural robots requires consistent maintenance and repairs. These ongoing costs and logistical demands can pose a significant burden, especially for smaller farms.
- Ethical issues and regulatory issues. (i) Regulatory regime: Adopting agricultural robotics requires close attention to both regulatory and ethical concerns. Regarding regulatory aspects, varying standards and requirements across different regions can create a complex landscape for compliance, potentially influencing the adoption and subsequent deployment of robotic systems. (ii) Ethics considerations: Bringing robotics into agriculture raises fundamental ethical concerns about farm labor, job security, and the wider impact on rural and remote communities. Addressing these ethical considerations is crucial to promote widespread acceptance and ensure the equitable distribution of the benefits offered by robotic technologies.

### 7.9 Ethical concerns

Ethical concerns in agricultural robotics center on data ownership, privacy, fairness, and the socio-economic impact of automation, especially in vulnerable farming communities. These issues require inclusive governance and responsible design.

1. Data sovereignty and privacy: The operation of agricultural robots generates highly granular data encompassing soil composition, crop health status, resource use (such as water and fertilizer), and minute details on farmer behavior and decision-making. A major ethical challenge arises because these valuable data often reside on proprietary cloud platforms owned by technology manufacturers. This setup raises serious concerns regarding data sovereignty: specifically, who legally owns, controls, and profits from the information generated on the farmer’s land. If these data are monetized or used for predictive modeling without explicit consent, farmers risk losing autonomy over their land and fundamental business decisions. As researchers note, “The most valuable harvest is no longer the crop, but the predictive pattern of the farmer’s decision-making,” making control over this information critical for maintaining farmer independence and fair market competition (Sustainability Directory, 2025).
2. Algorithmic bias and exclusion: It describes how inherent design flaws and training of AI-based systems can lead to inequitable outcomes and limit the benefits of agricultural robotics to only certain farmers.

This problem manifests in several critical ways:

Exclusion of smallholders: AI-driven systems require extensive data to function effectively. Because these systems are often trained on the rich datasets generated by large-scale, industrialized farms, they inherently favor these operations. As a result, smallholder and subsistence farmers with sparse or non-standard data are often excluded from the benefits and insights the technology provides.

Inequitable resource allocation: If the underlying training data contain bias, the resulting algorithms will perpetuate it. This can lead to inequitable resource allocation, where the system’s recommendations—for example, on irrigation schedules or pest control—systematically and unfairly favor certain plots or crop types over others.

Lack of local context: A reliance on generalized data means that algorithms often lack the local context necessary for accuracy. This lack of local knowledge on specific soil types, microclimates, and indigenous crops increases the risk of misclassification and poor decision support for farmers in various agroecological regions (Ribeiro, 2025).

1. Transparency and accountability: Transparency and accountability are essential ethical considerations in agricultural robotics, focusing on the clarity of how AI systems reach their decisions and the ability to assign responsibility when failures occur.

A core problem is that farmers often do not understand how decisions are made by robotic systems, especially when the outcomes are unexpected or contradictory to their experience. This lack of explainability means that if an error or malfunction occurs (e.g., misapplication of pesticides and crop damage), it is extremely difficult for farmers to contest the errors or hold developers accountable.

Without this transparency and a clear audit trail, a crucial element of trust is undermined, and farmers lose the ability to seek legal assistance when mishaps occur or the autonomous system malfunctions.

1. Labor displacement and economic disruption: The rise of agricultural robotics presents a significant challenge in terms of labor displacement and economic disruption because automation inherently reduces the demand for manual labor. This reduction, particularly in seasonal and physically demanding tasks, directly affects rural employment and the livelihood of agricultural communities that relying on such work.

To ethically deploy these technologies and prevent widespread hardship, there is a critical need for proactive strategies. Ethical deployment requires transition planning, upskilling, and inclusive innovation strategies to prepare the workforce for new, high-tech roles in the agritech sector. As noted by Sparrow and Howard, this issue extends beyond the mere use of technology: “The introduction of robots has political, social, and cultural implications that have received little attention,” underscoring the necessity of a holistic policy response to manage the shift in the labor market (Duckett et al., 2020).

1. Surveillance and autonomy: The integration of agricultural robotics introduces concerns about surveillance and farmer autonomy because of the comprehensive monitoring capabilities inherent in the technology.

Drones, ground robots, and networks of sensors are designed to continuously monitor farm activities from equipment movement to crop growth, creating an environment where every field action is logged and analyzed. This intense data collection raises significant ethical concerns about surveillance and privacy.

Furthermore, as these systems generate highly detailed data, they power algorithmic recommendations for planting, fertilizing, and harvesting. Farmers may feel increasing pressure to conform to these AI-driven dictates to maximize efficiency or qualify for certain insurance or supply chain programs. This reliance on the algorithm can gradually reduce the farmer’s agency and replace their experiential, intuitive decision-making with automated conformity.

## 8 The future of food: integrating robotics and sustainability

In general, advocacy for policy change is a powerful tool in promoting sustainable agriculture (Aninver Development Partners, 2025). Another perspective on the integration of robotics and sustainability is through policy recommendations. Developing countries must encourage the adoption of agricultural robotics by providing financial aid to farmers seeking to acquire robotic technologies for use in farm activities. Such assistance could take the form of grants, loans, and subsidies. In line with policy approaches, investment in research and development aims to make these technologies accessible and affordable through public–private partnerships and collaboration efforts. These partnerships can include awareness and community outreach, simplification and demystification of technologies, particularly robotics, offering complete training packages, capacity and capability development, and technical guidance to assist farmers in effectively utilizing harvesting robots (Aninver Development Partners, 2025). Policymakers must design regulatory regimes that are enabling in nature, minimize bureaucratic barriers, and provide tax credits to farmers investing in emerging technologies. Subsidies for the use of robotics, for instance, in the production of avocados, cassava, and fruits, combined with policies tailored to specific crops or regions, would generate significant value. In addition, providing financial and technical support to research and development institutions that focus on the adaptation and application of robotics in Africa can be crucial for overcoming barriers and realizing the full potential of this technology. Furthermore, the use of open-source forms helps advance the sustainability of agriculture—a case in point is that of LiteFarm (Jungblut, 2024). This open-source platform was developed from the ground up and is specifically tailored to the needs of sustainable farmers. Permarobotics, an innovative leader in digitalization to support regenerative agriculture, has donated the source code for its TraceFoodChain software to the Linux Foundation’s AgStack initiative. This open-source contribution represents a transformative step toward building more transparent, responsible, and sustainable agricultural supply chains (Alliance of Bioversity International and CIAT, 2025).

A summary of the impact of regional policies is presented in Table 7.

**TABLE 7:** Regional policy focus and recommendations for robotics in sustainable agriculture.

Region	Policy focus	Key recommendation
Southern Africa (Zimbabwe, South Africa, and Zambia)	Inclusive access and data sovereignty	Subsidize robotics for smallholders; establish rural repair and training hubs; align with national data protection laws; and promote open source platforms
South Asia (India, Bangladesh, and Nepal)	Agroecological adaptation and digital literacy	Develop zoning-based robotics standards; support PPPs for small farm robotics; and launch farmer digital literacy campaigns
Latin America (Brazil, Colombia, and Mexico)	Sustainability incentives and community inclusion	Offer green tech tax incentives; mandate ethical AI audits; and include indigenous communities in design processes
Europe (Germany, Netherlands, and France)	Interoperability and ethical design	Harmonize robotics standards; promote circular design and recyclability; and enforce explainable AI protocols
East Asia (China, Japan, and South Korea)	Smart farming zones and innovation acceleration	Establish smart farming pilot regions; create export compliance frameworks; and fund youth led robotics startups

The regional policy focus is broader, encompassing Southern Africa, South and East Asia, Europe, and Latin America. From a global perspective, the table outlines region-specific policy priorities for agricultural robotics, consistently emphasizing inclusive access, sustainability, and ethical innovation. Recommendations are highly tailored: Southern Africa focuses on subsidizing smallholder technologies and promoting open-source platforms, while Europe prioritizes the enforcement of explainable AI and circular design. This diversity reflects the differing developmental and governance contexts of each region. Fundamentally, these tailored strategies imply that successful digital transformation in agriculture requires localized standards, community engagement, and alignment with broader socio-environmental goals.

## 9 Conclusion

Food security generally implies that, at all times, all people have physical and economic access to sufficient, safe, and nutritious food that meets their dietary needs and food preferences for an active and healthy life. However, food security faces complex challenges, such as changing climatic conditions, resource shortages, and rapid population growth. Achieving adequate food security requires technological solutions that overcome the hurdles and attain agricultural resilience. This technological innovation takes the form of agricultural robotics, and its adoption helps address food security challenges. Food security is crucial as it impacts economic growth, public health, poverty reduction, sustainable development, and social stability. Through a blended mix of technology, sustainable practices, and relevant policy frameworks, food security can simultaneously be improved at both global and local levels. Decidedly, successful adoption of agricultural robots requires alignment of technological development with supportive frameworks and sustainable objectives.

This indicates that agricultural robotics is crucial to the eradication of hunger, as articulated in UN SDG 2. Zero hunger refers to the elimination of hunger, the achievement of food security, improved nutrition, and the promotion of sustainable agricultural practices globally. Despite the many promises demonstrated by the adoption of robotics, fundamental challenges such as investment costs, data security, and accessibility remain. To this end, further research is needed to overcome these challenges if global food security potential is to be realized in the near future.
